# Dental Bill

**Published:** 1868-02

**Authors:** 


### Editorial.

### DENTAL BILL.

A law pertaining to Dentistry has passed the House of Representatives in the Ohio Legislature, by a very satisfactory vote, there being sixty-seven yeas, and twenty-two nays. The bill is in a more acceptable form than has been before suggested. It is before the Senate, and we sincerely hope will receive its sanction. The proposed law is wholly prospective in its operation. The existence of such a law will be a great stimulus to the progress of the profession; it will not remove incompetency and quackery that now exists, but it will arrest its reproduction in the State.

